# LncRNA SNHG8 Promotes Proliferation and Inhibits Apoptosis of Diffuse Large B-Cell Lymphoma *via* Sponging miR-335-5p

**DOI:** 10.3389/fonc.2021.650287

**Published:** 2021-03-19

**Authors:** Bing Yu, Bo Wang, Zhuman Wu, Chengnian Wu, Juan Ling, Xiaoyan Gao, Huilan Zeng

**Affiliations:** ^1^Department of Hematology, The First Affiliated Hospital of Jinan University, Guangzhou, China; ^2^Emergency Department, The First Affiliated Hospital of Jinan University, Guangzhou, China

**Keywords:** diffuse large B cell lymphoma, apoptosis, long non-coding RNA, SNHG8, MiR-335-5p

## Abstract

Long-chain non-coding RNAs (LncRNAs) are expressed in diffuse large B-cell lymphoma (DLBCL) tissues and have played a regulatory role in DLBCL with a cancer-promoting effect. In this study, the role of LncRNA SNHG8 in the regulation of DLBCL cells is investigated, and its underlying mechanism is explored. The database of the Gene Expression Profiling Interactive Analysis (GEPIA) was searched, and the expression of SNHG8 in DLBCL and normal tissues was examined. The expression of SNHG8 was evaluated in several DLBCL cell lines and a normal lymphocyte cell line. It was found that SNHG8 was overexpressed in DLBCL tissues and cells in comparison with their normal counterparts. The short hairpin RNA (shRNA) plasmids of SNHG8 were transfected into DLBCL cells to knockdown the expression of SNHG8, followed by assays of proliferation, colony formation, apoptosis, and related protein expression. The results showed that the knockdown of SNHG8 significantly inhibited DLBCL cell proliferation and colony formation while promoting cell apoptosis. Moreover, the knockdown of SNHG8 reduced the expression of Ki-67, proliferating cell nuclear antigen (PCNA), and Bcl-2 and enhanced the expression of Bax and cleaved caspase 3/9. MiR-335-5p was predicted to be a potential target of SNHG8 by using the bioinformatics analysis, and the interaction between the two was validated by using the dual luciferase assay. In addition, the knockdown of SNHG8 increased the level of miR-335-5p, whereas miR-335-5p mimic decreased the expression of SNHG8. Finally, U2932 cells were co-transfected with or without sh-SNHG8 and miR-335-5p inhibitors, whose proliferation, colony formation, and apoptosis were determined subsequently. It was demonstrated that the presence of an miR-335-5p inhibitor partially canceled the inhibitory effects of the knockdown of SNHG8 on DLBCL cell proliferation and colony formation and the stimulating effects of the knockdown of SNHG8 on cell apoptosis. Taken together, our study suggests that lncRNA SNHG8 exerts a cancer-promoting effect on DLBCL *via* targeting miR-335-5p.

## Introduction

Being an aggressive malignant B-cell lymphoma, diffuse large B-cell lymphoma (DLBCL) is the most common subtype of non-Hodgkin lymphoma (NHL). DLBCL accounts for about 40% of newly diagnosed NHL cases in western countries (1). The lesions of DLBCL occur mainly on the lymph nodes, and some of them are found in the gastrointestinal tract, bone, and the central nervous system (2). The lymph nodes of patients with DLBCL enlarge rapidly at an early stage, and symptoms such as fever and night sweat can occur. With the progression of DLBCL, the lesions show aggressive growth, resulting in poor treatment outcome (3). Although the advancement of medicine contributes to a great progress in the therapeutic approach for the treatment of DLBCL, up to 40% of patients die from a relapse (4). So far, the molecular mechanism of the pathogenesis of DLBCL remains unclear, which seriously hinders the management of DLBCL.

Long-chain non-coding RNA (LncRNA) is a type of RNA that has a length >200 nucleotides. LncRNA was initially thought to be the noise of gene transcription because it does not have an open reading frame and rarely participates in encoding proteins. However, lncRNAs have been confirmed in recent years for the regulation of various diseases by binding to microRNAs or proteins, thereby participating in the occurrence, progression, and prognosis of diseases (5). The abnormal expression of lncRNA plays an important role in various cancers, such as hepatocellular carcinoma, breast cancer, prostate cancer, and non-small-cell lung cancer (NSCLC) (5–7). Therefore, it has been proposed that lncRNAs can become tumor biomarkers.

Long-chain non-coding RNAs also play a key role in the occurrence, progression, drug resistance, and prognosis of DLBCL (8). Verma et al. identified 2,632 novel multi-exonic lncRNAs that are expressed in more than one tumor. Two-thirds of the identified lncRNAs are not expressed in normal B cells, and one-thirds of them are differentially expressed in DLBCL subtypes (9). A study showed that lncRNA MALAT-1 is upregulated in DLBCL cell lines, and the downregulation of MALAT-1 inhibits the proliferation and migration of DLBCL cells, induces a cell cycle arrest, activates autophagy, and blocks doxorubicin-induced epithelial–mesenchymal transition, thereby reducing the doxorubicin resistance in DLBCL (10).

Long-chain non-coding RNA SNHG8 was demonstrated to have a cancer-promoting effect in various cancers such as lung cancer, liver cancer, colorectal cancer, pancreatic cancer, and endometrial cancer (11–13). However, the involvement of SNHG8 in DLBCL has not been reported.

The objective of this study is to investigate the role of lncRNA SNHG8 in DLBCL cells and uncover the potential mechanism.

## Materials and Methods

### Cell Culture and Treatment

Human B lymphocytes GM12878 and human DLBCL cell lines, including OCI-Ly10, OCI-Ly7, OCI-Ly3, and U2932 (ATCC, Virginia, USA), were cultured in RIMI-1640 medium (Thermo Fisher Scientific, Massachusetts, USA), supplemented with 10% fetal bovine serum (FBS) and 1% penicillin/streptomycin with an atmosphere of 5% CO_2_ at 37°C.

The short hairpin RNA (shRNA) targeting SNHG8 and negative scrambled control shRNA (sh-NC) were synthesized by GenePharma (Shanghai, China). MiR-335-5p mimic, inhibitor, and its NC; SNHG8 mutant (MUT); and wild type (WT) were obtained from GenePharma (Shanghai, China) and cloned into pcDNA3.1 vector. After reaching 80% confluence, cells were transfected with the indicated sh-RNAs, an miR-335-5p mimic or inhibitor, and SNHG8 MUT or WT using lipofectamine 2000 (Invitrogen, California, USA) according to the manufacturer's instructions. All transfected cells were then cultured for 48 h before the following experiments.

### Cell Counting Kit-8 Assay

Cells were seeded at a density of 3 × 10^3^ cells per well in a 96-well plate. After treatment with the indicated conditions for 24, 48, and 72 h, cells were incubated with 10 μl of the cell counting kit-8 (CCK-8) solution (MedChemExpress, MCE, New Jersey, USA) for 2 h at 37°C. The light absorbance of cells was measured at 450 nm using a microplate reader.

### Colony Formation Assay

For colony formation assays, cell suspension was resuspended in 1 ml medium. Samples were plated in a 24-well plate and incubated for 2 weeks. Crystal violet was used to stain cells, and a colony with >50 cells was counted as a colony.

### Real-Time Quantitative PCR

Total RNA was extracted using an RNeasy Mini Kit (QIAGEN, Maryland, USA) and reverse-transcribed into cDNA with SuperScript III First-Strand Synthesis SuperMix (Invitrogen, California, USA) according to the manufacturer's instructions. Quantitative PCR was performed by following the instructions of the HotStarTaq Master Mix Kit (QIAGEN). The reactions were performed in accordance with the following conditions: 95°C for 15 min, 25 cycles of 95°C for 15 s, 58°C for 30 s, and 72°C for 30 s, followed by 72°C for 10 min. The RT-PCR primers used were as follows:

SNHG8: forward, 5′-CCCGAGAACCGTCAGTTTGA-3′, reverse, 5′-ACACCCGTT-TCCCCAACTAC-3′;
MiR-335-5p: forward, 5′-TGTTTTGAGCGGGGGTCAAG-3′, reverse, 5′-TGAATAT-AGCAAATGAGAGG-3′; and
GAPDH: forward, 5′'-ACCTGACCTGCCGTCTAGAA-3′, reverse, 5′-TCCACCAC-CCTGTTGCTGTA-3′.

### Western Blotting

Total proteins from the cells were extracted by RIPA buffer with protease inhibitors (Beyotime Institute of Biotechnology, Jiangsu, China) on ice. Sodium dodecyl-sulfate–polyacrylamide gel electrophoresis (SDS–PAGE) was performed using equal amounts of protein samples for transferring the proteins to polyvinylidene difluoride (PVDF) membranes. On the first day, the membranes were incubated in 5% non-fat milk for 2 h at 37°C and then with primary antibodies against Ki-67, proliferating cell nuclear antigen (PCNA), Bcl-2, Bax, caspase-3/9, and GAPDH (1:1,000, Proteintech) at 4°C overnight. On the second day, the membranes were incubated with secondary antibodies (goat anti-mouse, 1:10,000; Proteintech) at 37°C for 2 h. Immunoblots were visualized by enhanced chemiluminescence (Santa Cruz Biotechnology, Texas, USA) and analyzed using ImageJ software.

### Flow Cytometry

Cell apoptosis was assessed by flow cytometry using the Annexin V-FITC Apoptosis Detection Kit (Beyotime, Shanghai, China). Briefly, cells were collected, washed with phosphate buffered saline (PBS), and then stained with APC-conjugated anti-Annexin V antibody and propidium iodide (PI) according to the manufacturer's protocol. The percentage of Annexin V^+^ PI^+^ cells was determined by using a flow cytometer (Becton Dickinson, New Jersey, USA).

### Luciferase Reporter Assay

Direct interactions between miR-335-5p and SNHG8 were predicted by Starbase 3.0 software (14). A luciferase reporter assay was performed by co-transfecting firefly luciferase reporter plasmids containing WT or MUT SNHG8 and renilla luciferase control reporter vectors (Promega, Wisconsin, USA) and the miR-335-5p mimic or miR-335-5p NC *via* lipofectamine 2,000 into U2932 cells. The luciferase assay was conducted at 48 h after transfection by using the Dual Luciferase Reporter Assay System (Promega, Wisconsin, USA) according to the previously described manufacturer's instructions (15).

### Statistical Analysis

The data are presented as the mean ± SD from three independent experiments. The Student's *t*-test was used for statistical comparisons between the two groups, and a one-way ANOVA was used for comparisons among multiple groups. A *p*-value < 0.05 was considered significant.

## Results

### SNHG8 Is Upregulated in DLBCL Cells

**Figure 1:**
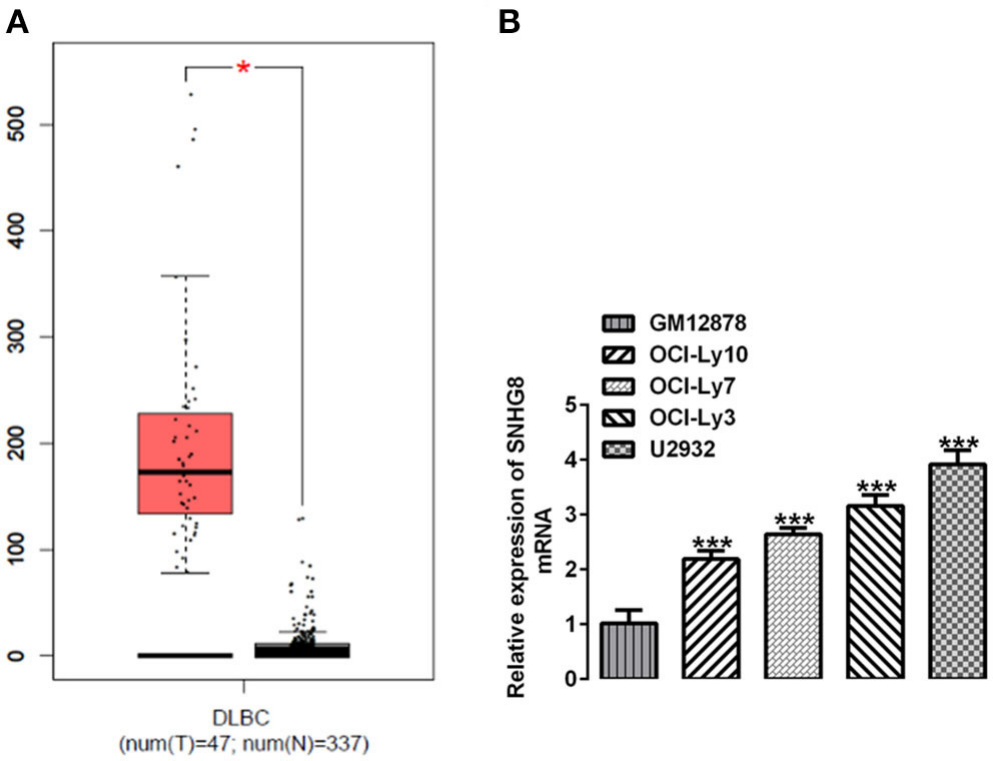
SNHG8 is upregulated in diffuse large B-cell lymphoma (DLBCL). **(A)** The levels of SNHG8 in DLBCL patients (left, red) and normal healthy individuals (right, black) and data from the Gene Expression Profiling Interactive Analysis (GEPIA) database. *p < 0.05. **(B)** The mRNA expression of SNHG8 in normal human B-lymphocytes, GM12878, and human DLBCL cell lines including OCI-Ly10, OCI-Ly7, OCI-Ly3, and U2932. ***p < 0.001 vs. GM12878.

We searched the database of the Gene Expression Profiling Interactive Analysis (GEPIA) and found that SNHG8 was significantly upregulated in the tissues of patients with DLBCL in comparison with that of normal healthy individuals (Figure 1A). We detected the mRNA levels of SNHG8 in normal human B lymphocytes, GM12878, and human DLBCL cell lines, including OCI-Ly10, OCI-Ly7, OCI-Ly3, and U2932. As shown in Figure 1B, the mRNA levels of SNHG8 in DLBCL cell lines were remarkably higher than those in GM12878. This result was consistent with the data from the GEPIA and suggested that lncRNA SNHG8 might play a role in DLBCL. Among these DLBCL cell lines, the U2932 cell line showed the highest level of SNHG8 and was, therefore, chosen for subsequent experiments.

### Knockdown of SNHG8 Inhibits Proliferation and Promotes Apoptosis of DLBCL Cells

**Figure 2:**
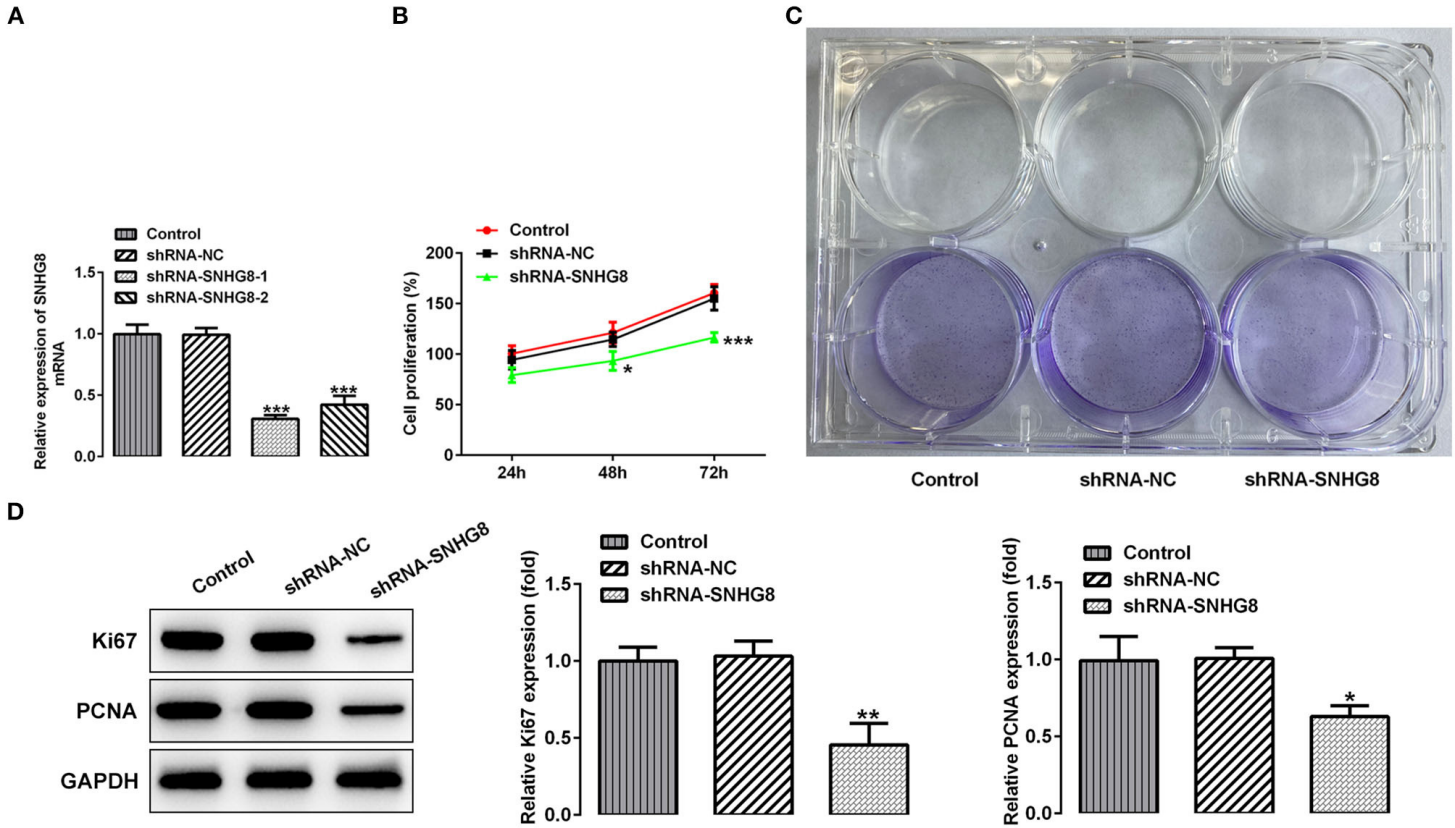
The effect of SNHG8 silence on cell proliferation of DLBCL. **(A)** The mRNA expression of SNHG8 in U2932 cells transfected with the indicated vectors. **(B)** The cell proliferation rate of U2932 cells transfected with the indicated vectors was measured by the CCK-8 assay. **(C)** Representative colony formation analysis for U2932 cells in different groups. **(D)** The protein expression of Ki67 and proliferating cell nuclear antigen (PCNA) in U2932 cells were detected by Western blotting. *p < 0.05, **p < 0.01, and ***p < 0.001 vs. short hairpin RNA of negative scrambled control (shRNA-NC).

To investigate the effects of SNHG8 on the cellular behaviors of DLBCL cells, two pairs of chemically synthesized shRNAs (shRNA-1 and shRNA-2) targeting SNHG8 and negative control (sh-NC group) were transfected into U2932 cells. The results from RT-qPCR showed that the expression of SNHG8 was inhibited by both shRNAs in comparison with the control and NC groups. The inhibitory efficiency of shRNA-1 was better than that of shRNA-2 (Figure 2A). Therefore, the cells transfected with shRNA-SNHG8-1 was chosen for subsequent experiments.

It was found that the cells that were transfected with shRNA-SNHG8 showed an obviously depressed cell proliferation in comparison with the cells that were transfected with shRNA-NC (Figure 2B). The results obtained from the colony formation assay also showed that the knockdown of SNHG8 inhibited DLBCL cell colony formation (Figure 2C). Moreover, the expression of proliferation-related proteins, Ki-67, and PCNA was significantly decreased by the knockdown of SNHG8 (Figure 2D).

**Figure 3:**
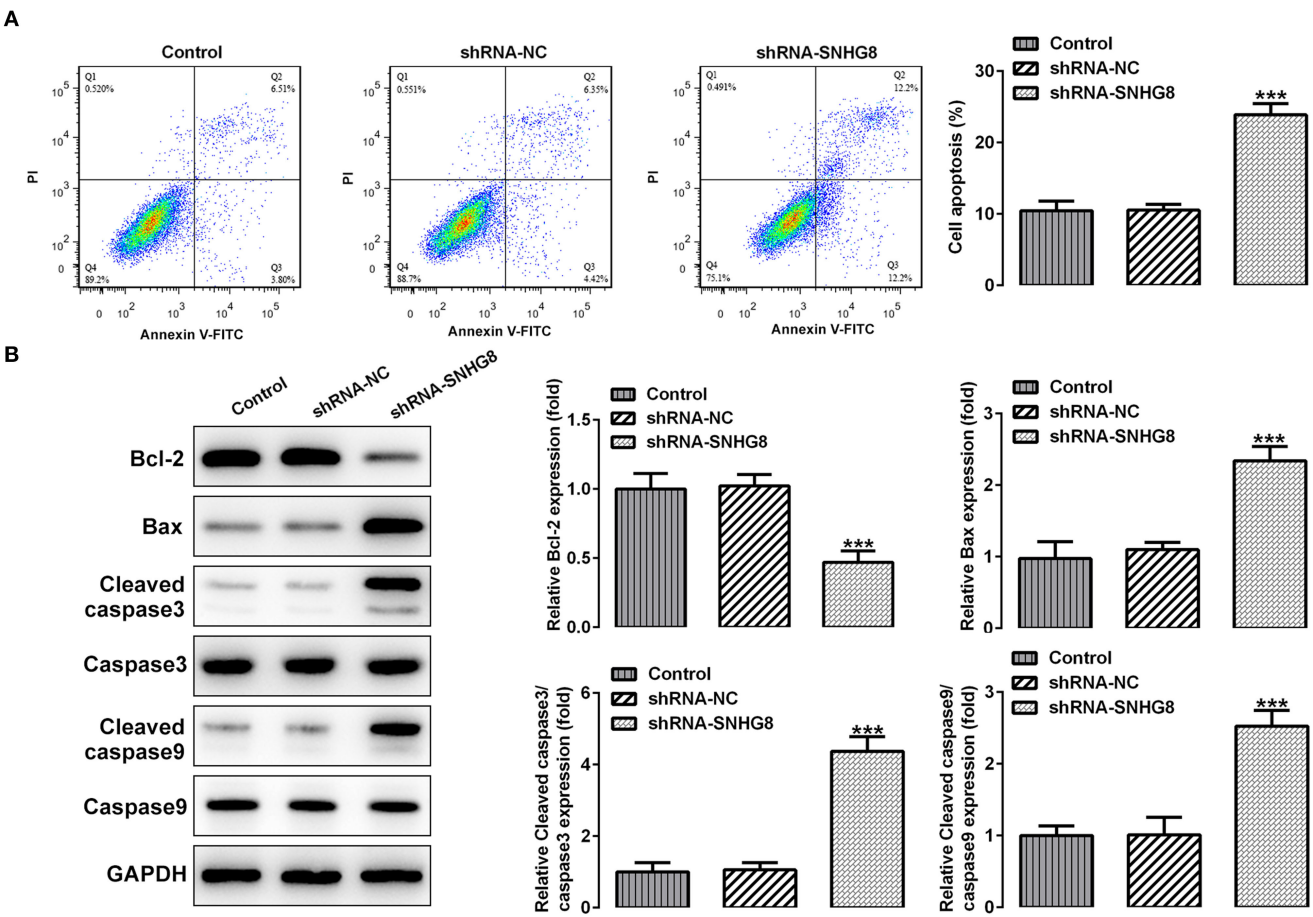
The effect of SNHG8 silence on cell apoptosis of DLBCL. **(A)** The apoptotic rate of U2932 cells with or without SNHG8 silence was measured by flow cytometry. **(B)** The expression of proteins involved in cell apoptosis in U2932 cells with or without SNHG8 silence were measured by Western blotting. ***p < 0.001 vs. shRNA-NC.

As shown in Figure 3A, the percentage of apoptotic cells was remarkably higher in cells that were transfected with shRNA-SNHG8 than the cells that were transfected with shRNA-NC. At the same time, a decreased expression of the anti-apoptotic protein Bcl-2 and an increased expression of pro-apoptotic proteins, such as Bax, cleaved caspase 9, and cleaved caspase 3, were observed in the shRNA-SNHG8 group in comparison with the shRNA-NC group (Figure 3B). These data indicated that the knockdown of SNHG8 suppressed the proliferation and induced apoptosis in DLBCL cells.

### MiR-335-5p Is Downregulated in DLBCL Cell Lines and Can Interact With SNHG8 to Affect Reciprocal Expression

**Figure 4:**
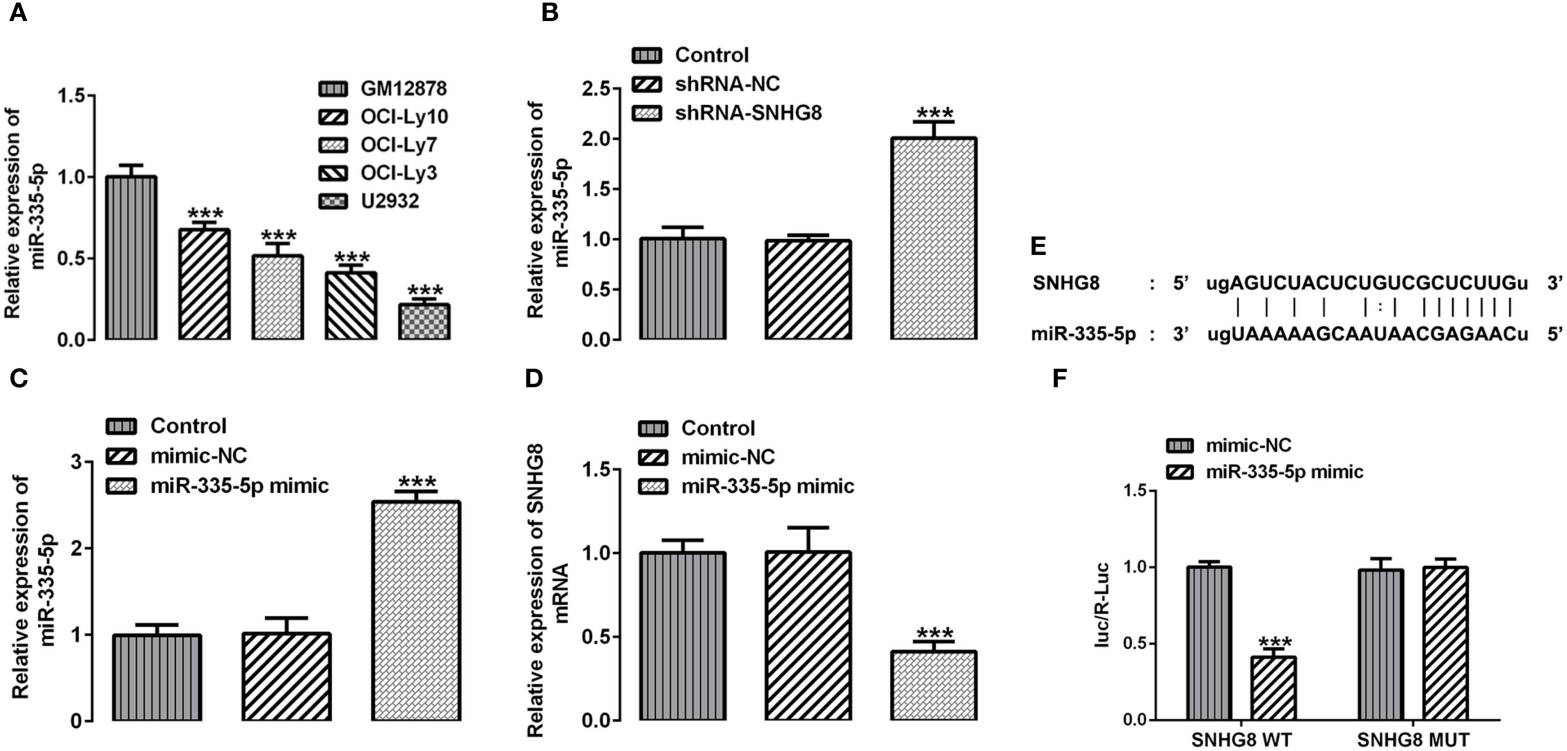
The interaction between SNHG8 and miR-335-5p. **(A)** The mRNA expression of miR-335-5p in normal human B lymphocytes, GM12878, and human DLBCL cell lines, including OCI-Ly10, OCI-Ly7, OCI-Ly3, and U2932. ***p < 0.001 vs. GM12878. **(B)** The mRNA expression of miR-335-5p in U2932 cells with or without SNHG8 silence. **(C,D)** The mRNA expression of miR-335-5p **(C)** and SNHG8 **(D)** in U2932 cells transfected with or without the miR-335-5p mimic. ***p < 0.001 vs. shRNA-NC. **(E)** The binding sites between SNHG8 and miR-335-5p. **(F)** Wild type (WT) or mutant (MUT) SNHG8 and the miR-335-5p mimic or miR-335-5p mimic-NC were co-transfected into U2932 cells, and then the luciferase reporter assay was performed to validate the direct binding between SNHG8 and miR-335-5p. ***p < 0.001 vs. mimic-NC.

With the help of the bioinformatics analysis using the StarBase software, we found that miR-335-5p might be a target of SNHG8. MiR-335-5p was reported to inhibit the invasion and metastasis of various cancers, such as thyroid cancer, colorectal cancer, and NSCLC (16–18). To evaluate the role of miR-335-5p in DLBCL cells, we examined the expression of miR-335-5p in the DLBCL cell lines. Contrary to SNHG8, the mRNA levels of miR-335-5p were significantly lower in DLBCL cell lines in comparison with normal human B lymphocytes GM12878 (Figure 4A). In addition, the silencing of SNHG8 increased the expression of miR-335-5p (Figure 4B), suggesting the interaction between SNHG8 and miR-335-5p in DLBCL cells. Then, we constructed the miR-335-5p mimic, which was transfected into U2932 cells. The upregulation of miR-335-5p was confirmed by qRT-PCR (Figure 4C). Further studies found that the overexpression of miR-335-5p reduced the expression of SNHG8 (Figure 4D). Moreover, WT or MUT SNHG8 and the miR-335-5p mimic or miR-335-5p mimic-NC were co-transfected into U2932 cells for the luciferase reporter assay. The result suggested the direct binding between SNHG8 and miR-335-5p (Figures 4E,F).

### Inhibition of the Expression of miR-335-5p Reverses the Effects of SNHG8 Knockdown on DLBCL Cell Proliferation and Apoptosis

**Figure 5:**
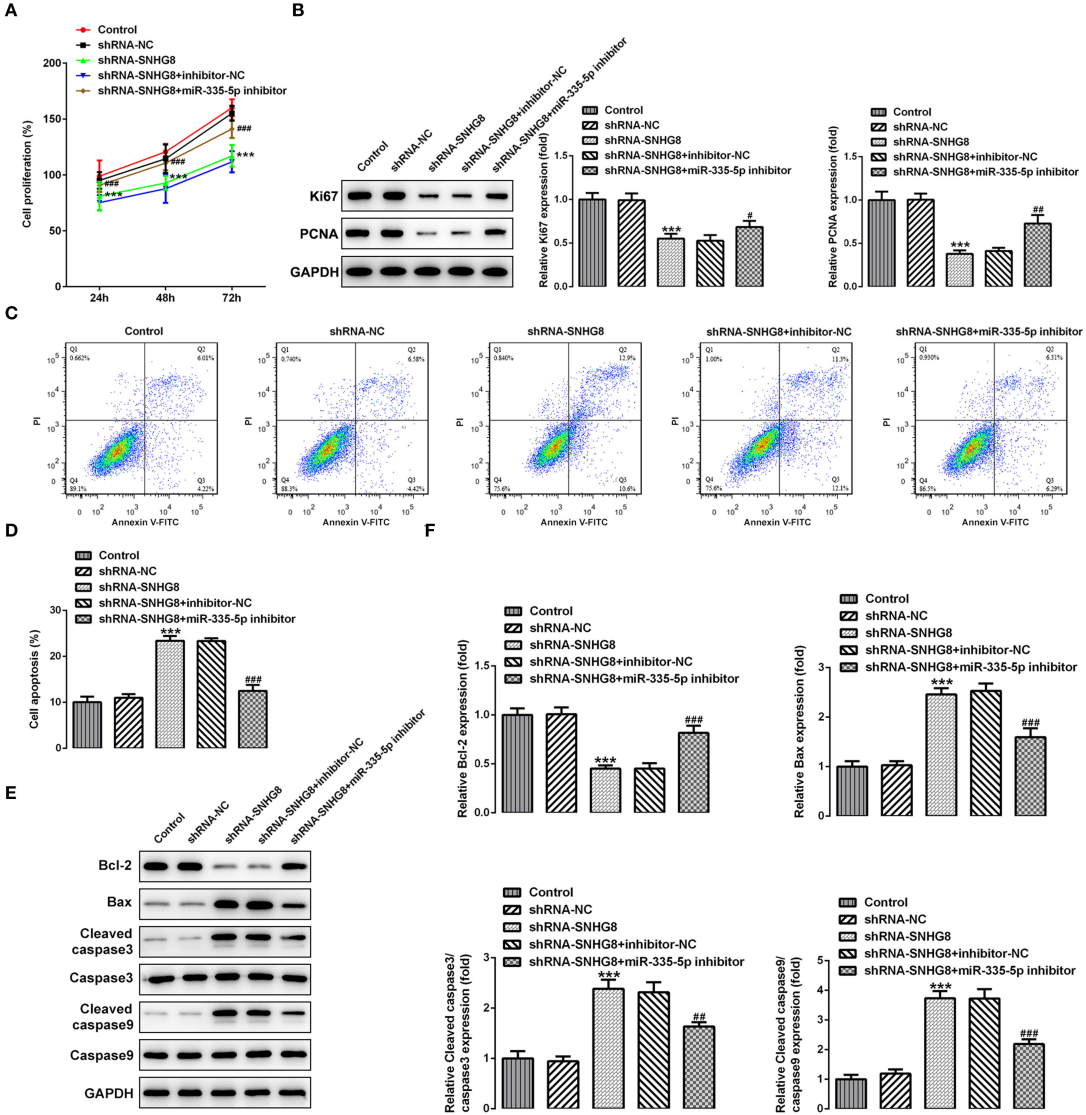
The inhibition of miR-335-5p blunts the effect of SNHG8 silence on DLBCL cell proliferation and apoptosis. **(A)** The proliferative rate of U2932 cells transfected with indicated vectors was detected by the CCK-8 assay. **(B)** The protein expression of Ki67 and PCNA in U2932 cells was detected by Western blotting assay. **(C,D)** The apoptotic rate of U2932 cells transfected with indicated vectors was measured by flow cytometry. **(E,F)** The expression of proteins involved in cell apoptosis in U2932 cells transfected with indicated vectors was measured by Western blotting assay. ***p < 0.001 vs. shRNA-NC. ^#^*p* < 0.05, ^##^*p* < 0.01, and ^###^*p* < 0.001 vs. shRNA-SNHG8 + inhibitor-NC.

Finally, determining whether the actions of SNHG8 were associated with the binding to miR-335-5p, cells with SNHG8 knockdown were exposed to the miR-335-5p inhibitor or inhibitor-NC. It was found that the cells co-treated with shRNA-SNHG8 and the miR-335-5p inhibitor exhibited a significantly higher rate of proliferation in comparison with the cells co-treated with shRNA-SNHG8 and the miR-335-5p inhibitor-NC (Figure 5A). Furthermore, the presence of the miR-335-5p inhibitor increased the expression of Ki-67 and PCNA (Figure 5B) and reduced the ratio of apoptotic cells increased by the knockdown of SNHG8 (Figures 5C,D). A Western blot analysis confirmed that the decreased expression of Bcl-2 and the increased expression of Bax and cleaved caspase 3/9 in cells with SNHG8 knockdown were partially reversed by co-treatment of the miR-335-5p inhibitor (Figures 5E,F).

## Discussion

Non-Hodgkin lymphoma has a high degree of heterogeneity and can occur in many sites of the body. DLBCL is the most common NHL pathological type, with an incidence as high as 30–40%. It is reported that DLBCL has a variety of histomorphology, clinical characteristics, and epigenetics (19). Usually, patients with stages I or II of DCBCL are treated with chemotherapy in combination with a comprehensive treatment, and those with stages III or IV of DLBCL are mainly treated with chemotherapy (20). The common chemotherapeutic drugs for DLBCL include methyl-benzylhydrazine, cyclophosphamide, nitrogen mustard, and other alkylating agents (21). The comprehensive treatment is to control distant metastasis of the tumor. Radiotherapy can effectively control the recurrence of local lesions and can cure about 50% of the patients (22, 23). However, the treatment efficacy in patients at an advanced stage is unsatisfied, and some patients are prone to relapse after treatment (24). Therefore, finding biomarkers that can detect DLBCL at earlier stages and identifying the risk factors for prognosis are the current focal points of the clinical management of DLBCL.

Long-chain non-coding RNAs can regulate the epigenetics, gene transcription, and gene post-transcription and play a significant role in the occurrence and development of tumors. LncRNA SNHG8 has been demonstrated to have a cancer-promoting effect in various cancers whose overexpression may be an independent risk factor for poor tumor prognosis (25). In addition, lncRNA SNHG8 can also be used as an early diagnostic biomarker for tumors (13, 25). So far, the role of lncRNA SNHG8 in DLBCL has not been elucidated. Therefore, this study takes the lead in analyzing the association between lncRNA SNHG8 and the pathological features of DLBCL.

Our results revealed an upregulated expression of SNHG8 in DLBCL tissues in comparison to the expression in healthy tissues and normal cell lines. Furthermore, the knockdown of SNHG8 could remarkably inhibit the proliferation and colony formation and promote the apoptosis of DLBCL cells, indicating that SNHG8 aggravates the progression of DLBCL and the knockdown of SNHG8 exerts an opposite effect. It is well-known that lncRNAs usually function as competing endogenous RNAs of specific microRNAs for the regulation of target genes. For example, SNHG8 has been identified as a key regulator in the progression of NSCLC *via* sponging miR-542-3p (11). Moreover, SNHG8 has been found to be upregulated in esophageal squamous cell carcinoma and to directly sponge miR-411 to increase oncogenicity (13). In this study, miR-335-5p was predicted by the GEPIA database to be a target microRNA of SNHG8 whose downregulation was also detected in DLBCL cell lines. Importantly, it has been demonstrated that miR-335-5p inhibits cell invasion and metastasis in thyroid cancer (18), a epithelial–mesenchymal transition in NSCLC (16), cell proliferation and metastasis in osteosarcoma (26), and cancer cell growth in colorectal cancer (17). Moreover, miR-335-5p acts as a target of lncRNAs to play a regulatory role in various cancers, such as osteosarcoma (27), cervical cancer (28), and bladder cancer (29). However, whether miR-335-5p regulates DLBCL remains to be investigated. In this study, we explored the interaction between SNHG8 and miR-335-5p and the role of miR-335-5p in DLBCL. Our results confirmed that miR-335-5p interacts with SNHG8 to regulate reciprocal expression. To further determine whether the actions of SNHG8 knockdown were dependent on an increased expression of miR-335-5p, we treated the cells with the miR-335-5p inhibitor in the presence of the knockdown of SNGH8. Our results demonstrated that the introduction of the miR-335-5p inhibitor significantly blunted the inhibitory effect of the knockdown of SNHG8 on cell proliferation and the stimulating effect of that on cell apoptosis. These results implied that SNGH8 sponges miR-335-5p to act on DLBCL. Furthermore, the effects of the miR-335-5p inhibitor on DLBCL suggested that miR-335-5p might inhibit the proliferation and promote the apoptosis of DLBCL cells. These data were inconsistent with the previous studies (16–18). However, the fact that the effects of the knockdown of SNHG8 were incompletely blocked by the miR-335-5p inhibitor, indicating the possible participation of other targets in the mechanism of SNHG8 in DLBCL. The mechanisms involved in the influence of miR-335-5p on cancers may be related to regulation of downstream signaling, such as ROCK1 and mitogen-activated protein kinase (MAPK) (30, 31), which was not clarified in this study. In addition, this study only focused on the *in vitro* cell model, lacking the validation of results from animal experiments. Therefore, further investigations on the above limitations shall be conducted in future studies.

In conclusion, the present study demonstrated that lncRNA SNHG8 can decoy miR-335-5p to facilitate the proliferation and inhibit the apoptosis in DLBCL cells. This study provides a better understanding of DLBCL and a novel therapeutic target for its treatment strategy.

## Data Availability Statement

The original contributions presented in the study are included in the article/supplementary material, further inquiries can be directed to the corresponding author/s.

## Author Contributions

HZ conceived the study and edited the manuscript. BY and BW performed the experiments and analyzed the results. ZW, CW, JL, and XG collected the samples and analyzed the results. BY wrote the first draft. All authors read and approved the final version of the manuscript.

## Conflict of Interest

The authors declare that the research was conducted in the absence of any commercial or financial relationships that could be construed as a potential conflict of interest.

## References

[1] Li S Young KH Medeiros LJ. Diffuse large B-cell lymphoma. Pathology. (2018) 50:74–87. 10.1016/j.pathol.2017.09.006 29167021

[2] Sukswai N Lyapichev K Khoury JD Medeiros LJ. Diffuse large B-cell lymphoma variants: an update. Pathology. (2020) 52:53–67. 10.1016/j.pathol.2019.08.013 31735345

[3] Ollila TA Olszewski AJ. Extranodal diffuse large B cell lymphoma: molecular features, prognosis, and risk of central nervous system recurrence. Curr Treat Options Oncol. (2018) 19:38. 10.1007/s11864-018-0555-8 29931605 PMC6294323

[4] Caro P Kishan AU Norberg E Stanley IA Chapuy B Ficarro SB. Metabolic signatures uncover distinct targets in molecular subsets of diffuse large B cell lymphoma. Cancer Cell. (2012) 22:547–60. 10.1016/j.ccr.2012.08.014 23079663 PMC3479446

[5] Fu D Shi Y Liu JB Wu TM Jia CY Yang HQ. Targeting long non-coding RNA to therapeutically regulate gene expression in cancer. Mol Ther Nucleic acids. (2020) 21:712–24. 10.1016/j.omtn.2020.07.005 32771923 PMC7412722

[6] Martens-Uzunova ES Böttcher R Croce CM Jenster G Visakorpi T Calin GA. Long noncoding RNA in prostate, bladder, and kidney cancer. Eur Urol. (2014) 65:1140–51. 10.1016/j.eururo.2013.12.003 24373479

[7] Gutschner T Diederichs S. The hallmarks of cancer: a long non-coding RNA point of view. RNA Biol. (2012) 9:703–19. 10.4161/rna.20481 22664915 PMC3495743

[8] Kang J Yao P Tang Q Wang Y Zhou Y Huang J. Systematic analysis of competing endogenous RNA networks in diffuse large B-cell lymphoma and Hodgkin's lymphoma. Front Genet. (2020) 11:586688. 10.3389/fgene.2020.586688 33193722 PMC7554339

[9] Jiang VY Du W Fairchild L Melnick A Elemento O. Transcriptome sequencing reveals thousands of novel long non-coding RNAs in B cell lymphoma. Genome Med. (2015) 7:110. 10.1186/s13073-015-0230-7 26521025 PMC4628784

[10] Li LJ Chai Y Guo XJ Chu SL Zhang LS. The effects of the long non-coding RNA MALAT-1 regulated autophagy-related signaling pathway on chemotherapy resistance in diffuse large B-cell lymphoma. Biomed Pharmacother. (2017) 89:939–48. 10.1016/j.biopha.2017.02.011 28292022

[11] Chen C Zhang Z Li J Sun Y. SNHG8 is identified as a key regulator in non-small-cell lung cancer progression sponging to miR-542-3p by targeting CCND1/CDK6. Onco Targets Ther. (2018) 11:6081–90. 10.2147/OTT.S170482 30275712 PMC6158002

[12] Song Y Zou L Li J Shen ZP Cai YL Wu XD. LncRNA SNHG8 promotes the development and chemo-resistance of pancreatic adenocarcinoma. Eur Rev Med Pharmacol Sci. (2018) 22:8161–8. 10.26355/eurrev_201812_16508 30556854

[13] Song H Song J Lu L Li S. SNHG8 is upregulated in esophageal squamous cell carcinoma and directly sponges microRNA-411 to increase oncogenicity by upregulating KPNA2. Onco Targets Ther. (2019) 12:6991–7004. 10.2147/OTT.S214881 31695414 PMC6717851

[14] Chen X Zhao S Li Q Xu C Yu Y Ge H. LncRNA NEAT1 knockdown inhibits retinoblastoma progression by miR-3619-5p/LASP1 axis. Front Genet. (2020) 11:574145. 10.3389/fgene.2020.574145 33281873 PMC7705249

[15] Wang QM Lian GY Song Y Huang YF Gong Y. LncRNA MALAT1 promotes tumorigenesis and immune escape of diffuse large B cell lymphoma by sponging miR-195. Life Sci. (2019) 231:116335. 10.1016/j.lfs.2019.03.040 30898647

[16] Du W Tang H Lei Z Zhu J Zeng Y Liu Z. miR-335-5p inhibits TGF-β1-induced epithelial-mesenchymal transition in non-small cell lung cancer *via* ROCK1. Respir Res. (2019) 20:225. 10.1186/s12931-019-1184-x 31638991 PMC6805547

[17] Zhang D Yang N. MiR-335-5p inhibits cell proliferation, migration and invasion in colorectal cancer through downregulating LDHB. J BUON. (2019) 24:1128–36. 31424671

[18] Luo L Xia L Zha B Zuo C Deng D Chen M. miR-335-5p targeting ICAM-1 inhibits invasion and metastasis of thyroid cancer cells. Biomed Pharmacother. (2018) 106:983–90. 10.1016/j.biopha.2018.07.046 30119270

[19] Liu Y Barta SK. Diffuse large B-cell lymphoma: 2019 update on diagnosis, risk stratification, and treatment. Am J Hematol. (2019) 94:604–16. 10.1002/ajh.25460 30859597

[20] Wong J Pickles T Connors J Parsons A Sehn L Freeman C. Efficacy of palliative radiation therapy (RT) for chemotherapy relapsed or refractory diffuse large B-cell lymphoma: a population-based retrospective review. Pract Radiat Oncol. (2020) 11:203–9. 10.1016/j.prro.2020.11.003 33197644

[21] Vercellino L Di Blasi R Kanoun S Tessoulin B Rossi C D'Aveni-Piney M. Predictive factors of early progression after CAR T-cell therapy in relapsed/refractory diffuse large B-cell lymphoma. Blood Adv. (2020) 4:5607–15. 10.1182/bloodadvances.2020003001 33180899 PMC7686887

[22] Tilly H Gomes da Silva M Vitolo U Jack A Meignan M Lopez-Guillermo A. Diffuse large B-cell lymphoma (DLBCL): ESMO clinical practice guidelines for diagnosis, treatment and follow-up. Ann Oncol. (2015) 26(Suppl. 5):v116–25. 10.1093/annonc/mdv304 26314773

[23] Van Den Neste E Schmitz N Mounier N Gill D Linch D Trneny M. Outcome of patients with relapsed diffuse large B-cell lymphoma who fail second-line salvage regimens in the International CORAL study. Bone Marrow Transplant. (2016) 51:51–7. 10.1038/bmt.2015.213 26367239

[24] Wang Y Farooq U Link BK Larson MC King RL Maurer MJ. Late relapses in patients with diffuse large B-cell lymphoma treated with immunochemotherapy. J Clin Oncol. (2019) 37:1819–27. 10.1200/JCO.19.00014 31170029 PMC7001527

[25] Liu J Yang C Gu Y Li C Zhang H Zhang W. Knockdown of the lncRNA SNHG8 inhibits cell growth in Epstein-Barr virus-associated gastric carcinoma. Cell Mol Biol Lett. (2018) 23:17. 10.1186/s11658-018-0070-8 29736176 PMC5924468

[26] Wang Y Zeng X Wang N Zhao W Zhang X Teng S. Long noncoding RNA DANCR, working as a competitive endogenous RNA, promotes ROCK1-mediated proliferation and metastasis *via* decoying of miR-335-5p and miR-1972 in osteosarcoma. Mol Cancer. (2018) 17:89. 10.1186/s12943-018-0837-6 29753317 PMC5948795

[27] Wang Y Yang T Zhang Z Lu M Zhao W Zeng X. Long non-coding RNA TUG1 promotes migration and invasion by acting as a ceRNA of miR-335-5p in osteosarcoma cells. Cancer Sci. (2017) 108:859–67. 10.1111/cas.13201 28205334 PMC5448616

[28] Liang H Zhang C Guan H Liu J Cui Y. LncRNA DANCR promotes cervical cancer progression by upregulating ROCK1 *via* sponging miR-335-5p. J Cell Physiol. (2019) 234:7266–78. 10.1002/jcp.27484 30362591

[29] Yang Y Wang F Huang H Zhang Y Xie H Men T. lncRNA SLCO4A1-AS1 promotes growth and invasion of bladder cancer through sponging miR-335-5p to upregulate OCT4. Onco Targets Ther. (2019) 12:1351–8. 10.2147/OTT.S191740 30863101 PMC6389014

[30] She JK Fu DN Zhen D Gong GH Zhang B. LINC01087 is highly expressed in breast cancer and regulates the malignant behavior of cancer cells through miR-335-5p/Rock1. Onco Targets Ther. (2020) 13:9771–83. 10.2147/OTT.S255994 33061456 PMC7533226

[31] Gao Y Wang Y Wang X Zhao C Wang F Du J. miR-335-5p suppresses gastric cancer progression by targeting MAPK10. Cancer Cell Int. (2021) 21:71. 10.1186/s12935-020-01684-z 33482821 PMC7821696

